# Ecological Psychology and Enaction Theory: Divergent Groundings

**DOI:** 10.3389/fpsyg.2020.00991

**Published:** 2020-05-26

**Authors:** Harry Heft

**Affiliations:** Department of Psychology, Denison University, Granville, OH, United States

**Keywords:** ecological psychology, enaction theory, perception theory, direct realism, information

## Abstract

Both ecological psychology and enaction theory offer an alternative to long-standing theoretical approaches to perception that invoke post-perceptual supplemental processes or structures, e.g., mental representations, to account for perceptual phenomena. They both do so by taking *actions* by the individual to be essential for an account of perception and cognition. The question that this paper attempts to address is whether ecological psychology and enaction theory can be integrated into a stronger non-representational alternative to perception than either one can offer on its own. Doing so is only possible if most of the basic tenets and concepts of ecological psychology and enaction theory are compatible. Based on an examination of the role that sensations play within each approach; the manner in which each treats the concept of information; and how each conceptualizes an organism’s boundaries, it is concluded that a synthesis of the two approaches is not possible. Particular attention is paid to the concept of sensations, the limitations of which were an impetus for the development of ecological psychology.

## Introduction

Most explanations of visual perception that have been offered in recent centuries, and in particular those following the tradition of British Empiricist philosophy, adhere to a common meta-theoretical template: sources of stimulation in the environment innervate sensory receptors, which give rise to elementary sensations that function as the basic components of perceptual experience. Because the character of the environment as experienced by the perceiver cannot be explained with reference to those sensations alone owing to their limitations, additional psychological processes beyond perception are deemed necessary that supplement, enrich, and organize them. These post-perceptual processes are assumed to be latent in the perceiver owing to inheritance or prior experiences or both. Since the emergence of cognitive psychology in the 1960s, these post-perceptual processes have often been claimed to be based on ‘mental representations’ of the environment.

The ecological approach to perception and enaction theory are distinctive among perceptual theories for advocating a theoretical approach that rejects an appeal to mental representations or post-perceptual processes to account for experience of the environment. Furthermore, both approaches assume that an adequate account of psychological processes other than perceiving, such as thinking, remembering, and communicating symbolically, requires as a first-step a satisfactory account of perceiving. For this reason, both agree that ‘getting one’s account of perceiving right’ from the outset matters a great deal for psychological theory broadly considered.

Because both ecological psychology and enaction theory attempt to establish a framework for a non-representational approach to psychological theory, and because presently both are in a minority position with respect to the field of perceptual psychology overall, it might seem as if a joining of forces as it were – or at least a partial synthesis – would make for a stronger joint alternative to the representational theories that have dominated psychology and the philosophy of mind for so long. That is only possible, however, if most of the basic tenets and concepts of ecological psychology and enaction theory – that is, their grounding concepts – are compatible. In this brief paper, I will argue that they are not.

Fultot et al. (2016) previously offered a comparative analysis of these two approaches and also reached a similar conclusion. The initial draft of the present paper was written prior to examining their analysis in order to develop an independent assessment. More importantly, since the appearance of Fultot et al. (2016), two major contributions to enaction theory have appeared: *Sensorimotor Life* (DiPaolo et al., 2017), and *Linguistic Bodies* (DiPaolo et al., 2018). The present paper draws mostly on the former as a basis for comparing ecological psychology and enaction theory.

It is recognized that not all investigators who self-identify as enactivists necessarily adhere to each of the features of the framework developed by DiPaolo and his colleagues. The purposes of this paper is not to survey the varieties of enactionism, however. Because that framework bears most of the conceptual characteristics of this approach overall, and because at present it appears to be the most influential in addition to the seminal work by Varela et al. (1991), this paper focuses primarily on those three books.

## Starting Points

In order to examine the areas of difference between ecological psychology and enaction theory, it will be useful here at the outset to recognize that the starting points in the formulation of these two approaches differ. On the one hand, ecological psychology is rooted in James Gibson’s account of perception which emphasizes the role that perceiving plays for the organism in the control of action, and conversely, the significance of action in the organism’s detection of properties of the habitat (Gibson, 1958, 1966, 1979). Perceiving supports adaptive functioning by making it possible for the organism to ‘stay in touch’ with the environment in the course of everyday actions. The formative image underlying enaction theory, on the other hand, is the living cell operating as a far-from-equilibrium, dynamic system that strives to maintain stability in the face of possible perturbations (DiPaolo et al., 2017, 2018). It does so by way of its network of interdependent within-system processes and through continuous exchanges with the surround beyond its boundaries.

It is the case that these differences in their starting points can ultimately be reconciled. Adherents of each approach have independently argued how ecological psychology and enaction theory considered on their own terms are compatible with dynamical systems thinking (e.g., Chemero, 2009; DiPaolo et al., 2017, respectively). But reconciling them in this regard will not be sufficient for their rapprochement. This is because the lines of thought in ecological psychology and enaction theory beginning with their most basic concepts led to noteworthy divergences between them. Reconciliation would require non-trivial modifications in the conceptual structure of one approach or the other. A simple melding of the two will not do.

It is also critical to emphasize here at the outset that a central feature of the framework developed from the ecological psychology perspective – indeed, its essential commitment – is one that would *not* be embraced by enaction theory. Advocates of ecological psychology maintain that their approach to perception provides grounds for the claim that the environment is *directly perceived.* Direct perception means that perception of the environment – that is, the detection of its relational structure by means of perceptual systems – is not mediated by non-perceptual processes such as stored memories, mental representations, and the like. Ecological psychology offers a conceptual basis for embracing the epistemological position of *direct realism* (Gibson, 1967). When enaction theory extrapolates the image of the living cell as a dynamic, far-from-equilibrium system to an account of perception, it also sees no need to appeal to stored memories and mental representations. In spite of that, as we will see, one would be hard-pressed to describe its account of perceiving as direct, nor would one characterize its epistemology as that of direct realism. This difference will emerge at several points below.

The present discussion will mostly be limited to considerations of visual perception. In order to explicate their areas of disagreement, it will be necessary to review what may be quite familiar ground for some readers. Doing so is intended, in part, to inform those committed to one or the other approach about its counterpart. While my own training stems from an ecological approach, I trust that proponents of enaction theory will consider my account of their views as being accurate as far as it goes.

Three points of difference between the two approaches will be discussed here: (1) the role that *sensations* play within each approach; (2) the manner in which each approach treats the concept of *information*; (3) the way each approach conceptualizes an *organism’s boundaries*. To some extent, these differences hinge on matters of terminology and the way particular concepts are defined. But these terminological and definitional differences are far from trivial. They are indicative of fundamentally dissimilar approaches to perception.

In brief, ecological psychology characterizes perceiving on the part of the individual as a process of perception-action involving the pickup of *information* in the environmental surround that is *available* to the perceiver and that *specifies* properties of the environment. Enaction theory claims that the perceived environment is realized, comes into being, is ‘enacted’ for an individual by means of an interdependent dynamic network of *sensorimotor* processes within the boundaries of the organism. Obviously, these two claims require a great deal of elaboration, but they are sufficient as places to begin our discussion because they bring to the foreground a few notable differences in terminology employed in each approach. Ecological psychology takes as a core concept ‘information’; whereas central to enaction theory is the concept of ‘sensorimotor processes.’ As we will see, each concept as used in the respective theories would be rejected by its counterpart approach on grounds to be explained. The differences between ecological psychology and enaction theory could not be more clearly revealed than when we compare their respective treatments of information and sensorimotor processes.

## The Role of Sensations in Perception

As already discussed, the standard approach to explaining perception takes sensations as its starting point and develops an account of perceptual processes from there. The ecological psychology concept of information, and ultimately the ecological approach to perception itself, was developed by James Gibson in large measure *because* he came to the realization after many years that a functionally adequate account of perception – that is, one that describes the process by which organisms function in the environment in the course of everyday activities – could not be formulated based on what are conventionally taken to be ‘sensations.’ That point cannot be overemphasized. To the extent that sensation in this conventional sense corresponds to how the term is employed in the expression ‘sensorimotor’ in enaction theory, this difference sets ecological psychology and enaction theory on diverging paths from the outset.

### Conceptual Limitations of Sensations From the Standpoint of Ecological Psychology

Following Turvey (2019, pp. 166–167), sensations are conventionally assumed to have the following characteristics: they are *anatomically specific* products of sensory receptor stimulation, and as such they are *biological correlates* of physical energy variables originating in the environment. As biological correlates of receptor stimulation, they are *private*, occurring ‘in’ the organism. Importantly, owing to their origins in individual receptor functioning, sensations are assumed to be *discrete* as well as *transient*.

In contrast, perceptual experience tends to have the qualities of *patterns* and ordered or semi-ordered *structure* rather than discrete bits of sensation. Further, features of perceptual experience, such as objects, tend *not to be transient*: even when they *go out of sight* they usually are not experienced as *going out of existence* [see Gibson (1979) treatment of dynamic occlusion (Chap. 11); Heft (2020)]. They have a phenomenal permanence to them (excepting somewhat less common cases such as disintegration of matter and evaporation of liquid.) Finally, features of perceptual experience typically are ‘felt’ to be located in *a public domain* beyond the body boundaries – and as such, they are taken to be qualities that, in principle, others can experience as well, rather than being exclusively private.

The recurring challenge for perceptual theorists has been how to explain this apparent ‘gap’ between properties of sensations, on the one hand, and perceptual experience, on the other. Ecological psychology and enaction theory offer alternative accounts. Enaction theory offers an account of perceiving whereby system processes incorporate sensations into a sensorimotor loop, by means of which perceptual experience of the environment is realized (‘enacted’). Ecological psychology, in contrast, rejects the assumption that sensations play a role in perceiving; instead they are considered to be incidental to perceptual experience. Instead of a sensation-based account, ecological psychology offers an ‘information-based account of perceiving.’ That is, ecological psychology, unlike enaction theory, dispenses with sensations in its account of perceiving. What is directly perceived is the environment. For this reason, the proximal-distal distinction found in most modern accounts of perception collapses. Although this step is unorthodox among theories of perception, it is not entirely novel, having been previously proposed by Reid (1785) and James (1890, Chap. 17).

### Enaction Theory and Sensations

In their seminal book for enaction theory, Varela et al. (1991) hold that “the enactive approach consists of two points: (1) perception *consists in* perceptually guided action and (2) cognitive structures emerge from *recurrent sensorimotor patterns* that enable action to be perceptually guided” (p. 173, emphasis added). Perceptual experience, that is, is a resultant of linkages between sensations and motor activities rather than solely based on the deliverances of sense. They appeal to sensorimotor patterns rather than sensations as such, not because of the characteristics mentioned above, but because sensations change in the course of on-going activity.

The point of departure for the enactive approach is the study of how the perceiver can guide his actions in his local situation. *Since these local situations change as a result of the perceiver’s activity*, the reference point for understanding perception is the sensorimotor structure of the perceiver (the way the nervous system links sensory and motor *surfaces)* (Varela et al., 1991, p. 173, emphases added.)

For that reason, perception necessarily must stem from action patterns in relation to sensations, or a network of sensorimotor linkages.

But can sensations, even when they are embedded in a sensorimotor loop, carry correlates of environmental structure that are sufficiently ‘informative’ about the nature of the environment so as to allow for adaptive functioning? In other words, can sensations as conventionally understood (see above) carry the conceptual weight needed for an account of perceiving the world *beyond the system’s boundaries*? Even allowing for the possibility that perceptual experience is dependent on sensorimotor linkages ‘in’ the system, the sensations must carry *some* qualities of the environment beyond the system’s boundaries so that the organism is not ‘free-floating’ wholly detached from the surround. While a sensorimotor account may address how a system itself strives to maintain stability, does it also allow for a means by which the organism can stay in touch with and anchored to the environment? (How the environment is conceptualized is a related contentious issue, as we will see below).

Recognizing that the question of what makes a particular sensorimotor pattern informative requires attention to other aspects of their framework to be taken up later, here we focus on the ‘sensory’ part of the sensorimotor loop. In that regard how do enaction theorists describe the character of the ‘sensory’ facet of a sensorimotor structure? As far I have been able to determine, the qualities of sensations are rarely described with much specificity in the enaction theory literature. To the extent that they are, they would appear to be referred to as the products of sensory receptor stimulation. DiPaolo et al. (2017) describes the sensorimotor approach as taking “*the raw and quantifiable variation* of sensory and motor surfaces of the organism as a departure point” (p. 32, emphasis added). They continue that the focus is on the way in which ‘the *sensory stream* changes’ with movements of the agent; on how “the agent guides its movements in relation to *sensations*” (p. 34), and with “co-variations of *sensory stimulation*, neural, and motor activity” (p. 43; emphases added). As this sampling shows, references to ‘the sensory stream’ in enaction theory writings are rather general in nature. If the ‘sensory’ in sensorimotor activity is intended to refer to something other than sensations in a conventional manner, namely, as the products of receptor activity, then it is incumbent for enaction theorists to be more specific here.

Perhaps the most detailed account of the experience of sensations offered in the initial chapters of DiPaolo et al. (2017) concerns the hypothetical case, previously suggested by Myin (2003), of how an individual might identify by touch alone the property of the sponginess of “a small spongy ball. [when it] is held between thumb and forefinger.” Squeezing the ball (an action) produces a felt pressure on the finger tips, as well as “propriception, and the sense of effort required to maintain a certain grip” (p. 58). As we will see in the next section, this felt pressure produced through action occurs in relation to background knowledge of other possible sensations that *might* arise in this case. Here we limit our attention to sponginess as a ‘proximal property’ stemming from the immediate contact between a source of stimulation and receptor surfaces. As a proximal property, the experience of sponginess does not ‘reach’ distally into the surround beyond the body boundaries. The fact that the object is even a *ball*, for example, is not discernible based on that sensation, obviously.

Other examples of sensations offered include the flow of stimulation that results from movement relative to the environment. If, for example, I move my eyes, sensory patterns due to light projecting on the retina sweep, albeit discontinuously due to saccades, across the retinal surfaces. The flow of stimulation can only be determined to be contingent on movement owing to the correspondence between sensory and motor effects; but the sensory stimulation itself, even in the context of a sensorimotor loop, provides no ‘information’ *about the environment*. All that one can discern is a *proximal* retinal flow and whether or not the perceiver herself caused it. It has no ‘distal’ referent.

DiPaolo et al. (2017) write: “The primary correlation that is available to an agent is the manner in which the sensory stream changes as a function of its own actual movement and its possibilities and dispositions for movement.” They continue: “From this perspective, agency is about enacting effective sensorimotor relations. *These are the relations that the agent helps to create and which are immediately available to it*” (p. 32; emphasis added). It is by means of a mastery of sensorimotor regularities that one comes *to perceive the world* and the self, or in their phrasing, one engages in “sense-making.”

But is the world beyond proximal sources of stimulation even accessible to the perceiver in such an account? To return to the examples, sponginess versus solidity, or retinal flow produced through self-motion are, at best, proximal experiences – qualities that are limited to the immediate contact of physical stimulation and sensory receptors. But perceptual experience is much more than that. We experience a world that surrounds and extends ‘away’ from us. That is, we have ‘distal’ experiences. The evolution of vision (as well as audition and olfaction) quite likely is due to the functional value of detecting features of the environment at a distance from the perceiver. The language of ‘sensations’ would seem to trap enaction theory within the dynamic system that is the organism. Experience of the environment is claimed to be realized by way of sensorimotor linkages, but how is that realized experience connected to the environment as such?

To get beyond system boundaries involves, as we saw above, what enaction theorists call sense-making – the enactment of the perceived world. That may be assumed to take the perceiver beyond proximal ‘contact’ with the world; but ultimately the ‘distal’ sources of sensations would seem to be conjured up by some means other than ‘direct’ contact because sensations are inadequate to do the necessary work. It is for this reason enaction theory has the appearance to some critics as being a form of Idealism, although its proponents would surely reject that attribution because it would seem to remove the approach from the realm of natural science.

From the point of view of enaction theory, the preceding criticism concerning the limitations of sensations might well be viewed as a ‘straw man’ argument, because the enaction approach invokes sensorimotor networks rather sensations alone. But will sensorimotor networks overcome the conceptual limitations of sensations? What perceptual work revealing the world beyond the body boundaries is the *sensory facet* of sensorimotor networks supposed to able to contribute? Absent a more detailed account of what is meant by the products of sensory stimulation, it appears to be an exceptionally impoverished concept on which to build an account of perception of *the environment* even after embedding them in the notion of a sensorimotor structure. Recall that it was because of such conceptual limitations of sensations that Gibson turned away from them in his efforts to develop an account of perceiving. The ecological approach to perceiving is built on entirely different grounds. We will sketch that out next, and in doing so compare how the approaches employ the notion of information.

## Information for Perceiving

### Ecological Optics: Information-as-Specification

At the heart of an ecological approach to visual perception is Gibson’s proposal and exposition of *ecological optics*. It appears to me, at least, that Gibson’s framework is often misunderstood because ecological optics is not given adequate attention by commentators, including those working from an enaction perspective. Gibson (1966) envisioned ecological optics as a piece of an overall, and still developing, ‘ecological physics’ which considers the physical energetic properties of the world *relative to active organisms taken as a whole*, rather than in relation to the more reductive level of sensory receptors. In other words, he is attempting to offer a description of the environment in relation to animal life rather than the world from the standpoint of an animal-free domain of physics. Gibson saw the historical inclination of philosophy and psychology to begin the consideration of perception with terms developed within conventional physics, and in turn with the stimulation of receptor cells giving rise to sensations, to be the basis for many enduring theoretical and philosophical problems in psychology (Reed, 1988; also see, Dreyfus and Taylor, 2015).

To be more specific, an ecological approach to the study of visual perception among terrestrial organisms begins not with a micro-consideration of light in a classical physical vein (e.g., light traveling in waves of different periodic frequencies and intensities or as photons), but instead with a consideration of the illuminated environment taken at the level of the active organism as a whole – that is, with a consideration of the *habitat*. From an ecological/evolutionary stance, animals adapt to their habitat as whole organisms, not merely piece-meal (Lewontin, 2000). For this reason, an examination of the ecological possibilities for perceiving the habitat should be taken at a level of analysis commensurate with the organism considered as a whole. Broadly considered, then, the habitat for a terrestrial species includes the ground surface layout; detached and attached objects on those surfaces; and events transpiring over a perceptible duration (see Gibson, 1979, Chap. 3).

Ecological optics is an on-going research field that considers how light from a radiant source (e.g., the sun) interacts with surfaces, such as inanimate and animate features and the ground. When surfaces are illuminated, some of that light is absorbed, and some is reflected by them. Owing to such things as the reflectance character of surfaces due to, e.g., pigmentation and texture, as well as their orientation relative to the light source, *reflected light* takes on some of the character of these surfaces. To offer a simple example, white surfaces reflect more light than dark surfaces; and surfaces perpendicular to the ‘lines’ of light radiation reflect more light than those oblique to them. Adjacent perpendicular and oblique surfaces produce a discontinuity in reflectance or an edge. As these simple cases illustrate, reflected light is rarely homogenous, but structured even in these minimal ways. The continuous, instantaneous scattering of reflected light due to the abundance of surfaces present in most places results in a ‘steady state’ of reflected light intersecting at, in principle, an infinite number of potential observation points. These potential observation points can be temporarily occupied by a perceiver, and more commonly, individuals move along a path of observation points (Gibson, 1966). The scattering of reflected light is *ambient* with reference to an individual: it surrounds the individual, rather than being considered solely as light rays that project onto a picture plane (i.e., the retina) as it is in standard accounts of visual perception.

A detailed analysis of the structure of reflected light *to a particular point of observation* shows that some properties of the terrestrial environment and its features, such as surfaces that extend away from the viewer and the perception of relative object size, can be carried in or conveyed in the structure of reflected light (Gibson, 1950). When the structure of reflected light is considered *from a moving point of observation*, rather than from a single observation point, information specifying object shape and self-motion become available to a potential perceiver. Detailed accounts of ecological optics can be found in numerous sources, such as Gibson (1966) and Sedgwick (1986) On-going research considers various abstract geometric systems that might be best utilized to describe patterns of reflected light to an observation point (see, Warren, 2020).

The structure in ambient light that *specifies*, e.g., surface layout, is referred to by Gibson as *information*. Information in the available array of reflected light corresponds to or *specifies* properties of the habitat, and is often most readily detected from a moving point of observation. Through the detection or ‘pick up’ of this structure (sometimes the metaphor ‘resonate’ is invoked), organisms perceive the layout of the habitat. In order to distinguish this use of ‘information’ from how the term information is employed in other psychological theories (see below), we will refer to this as ‘information-as-specification’ following Turvey (2019).

Let us consider in a bit more detail the manner in which structure in ambient light can function as specifying information. Gibson distinguishes between *invariant structure* and *perspective structure* in the ambient array of light, both of which are revealed from the point of view of an active perceiver. Also, invariant structure can be detected from a temporary stationary position, with, e.g., displacements and rotations of objects. *Invariant structure* refers to those patterns in ambient light that do not change in the context of change. For example, the perceived shape of an object is experienced as remaining constant even as one walks around it; and this constancy can be attributed to invariant relationships in the reflected light. These invariant relationships that are perceived over time remain the same across changing points of observation, and they have been have been described geometrically for several cases (see Johansson, 1964; Johansson et al., 1980; Runeson, 1994).

*Perspective structure* is the flow of reflected structure in the ambient array of light that is generated by a moving perceiver in relation to surface layout when adopting different but continuous points of observation (e.g., optic flow). In the previous example, the visual experience of oneself moving around the object is conveyed by perspective structure. Movement toward an object is specified by an optic flow of structure, and parameters within that flow specify the direction and rate of movement. A great deal of supportive research has been carried out to describe mathematically the changing structure of reflected light from a moving point of observation (e.g., Lee, 1976; Johansson et al., 1980; Warren, 1998; Fajen, 2005).

### Sensation-Less Perception

**FIGURE 1:**
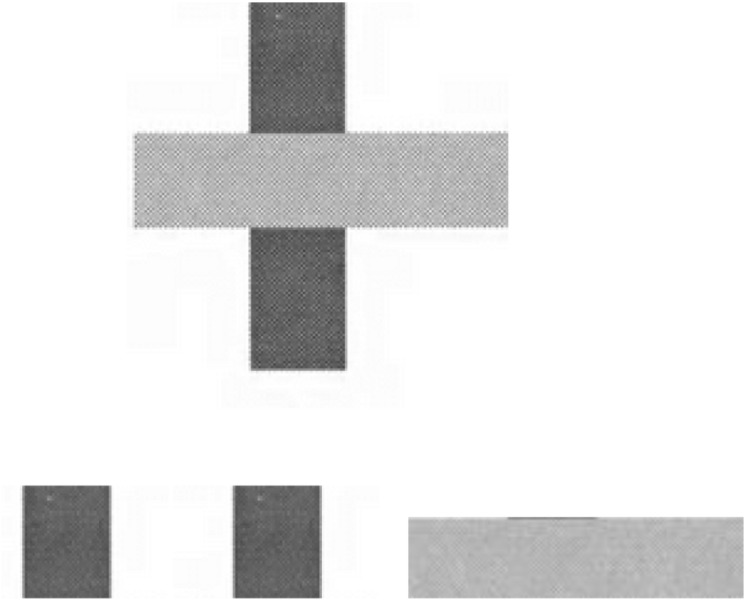
Figural occlusion.

An especially vivid contrast between an approach to perceiving based on sensations and an information-based approach are cases of so-called ‘sensation-less perception.’ Here we can consider if sensory stimulation is even a necessary constituent of perceiving in all cases. Gibson (1966, 1979) has argued that it is not, and in support of this assertion he offers instances when features of the environment are perceived in spite of the fact that those features cannot give rise to any sensations because those features are temporarily out of sight. Take, for example, instances when objects or portions of objects are temporarily hidden from view, such as when an object visually occludes the surface of another object in the line of sight. Figure 1 is a pictorial representation of such a case. (Because of its static nature, Figure 1 is merely suggestive of an effect that is readily experienced dynamically; see below.) A horizontal bar juxtaposed over a vertical bar is not typically described by perceivers as three separate objects, but rather two objects, with one partially occluding another. The partially occluded surface is typically experienced as a single vertical bar as if the surface that is presently occluded *persists* even though it cannot be fully viewed from one observation point – there are no sensations specific to these hidden portions of the surface given the current line of sight. Consistent with these observations are studies that show young infants will track the position of an object as it passes behind an occluder (Gibson and Pick, 2001, pp. 122–125; also see Van der Meer and van der Weel, 2020). Their actions suggest an awareness of its *persistence* in experience even though there are no sensations possible when the object is passing behind the occluder.

How are these phenomena to be explained from an ecological perspective? Recall that the ecological approach claims that perceiving is a process of detecting information. Is there information that specifies a surface going out of sight (as opposed to going out of existence)? When a perceiver moves relative to the two object surfaces located at different distances from him but in the same line of sight – or alternatively, when one surface moves relative to the other, e.g., an animal passing behind a tree – what occurs is a gradual ‘covering up’ over time of once visible portions of the occluded object at the ‘leading edge’ of the occluding object; and a gradual ‘revealing’ over time of once hidden portions of the occluded edge at the ‘trailing edge’ of the occluding one. (Refer to an experimental film made by Gibson to demonstrate of this compelling effect dynamically from a fixed viewing position^1^.) The event of gradual occlusion/disocclusion of the more distant (occluded) surface at the leading/trailing edge of the closer (occluding) surface is information in the ambient reflected light of, respectively, portions of the more distant object temporarily going out of sight, while other portions once out of sight are revealed. This information is only perceivable over time; and notably, for our discussion of sensation-less perception, those portions of the more distant object that are currently out of sight are experienced as persisting. The important point for our purposes here is that there is perception (experience/awareness) of the persistence of the occluded object in the absence of sensations.

Although I have not found reference to the occluding edge phenomenon in the enaction literature, it could be accounted for within their approach as well. And yet the manner in which they would approach this phenomenon surely would be quite different from that of ecological psychology. These differences illuminate how the two approaches are at variance. Presumably from the point of view of enaction theory, because of learned (or perhaps innate) contingencies linking sensory and motor patterns – that is, because of the sensorimotor loop that is in place – the sensorimotor system ‘enacts’ or gives rise to the perceptual experience of a complete vertical bar partially occluded from view by the closer horizontal bar. This would be an instance of the ‘sense-making’ possibilities of sensorimotor processes. Because it is assumed that sensations are constituents of perceiving, and because in the case of an occluded object there are no sensations available, that which is out of sight presumably must be *enacted* via sensorimotor processes.

One may argue that both accounts amount to much the same thing – and in terms of outcome this may be the case. Both attempt to explain how it is that a perceiver can be aware of an object or a portion of an object presently out of sight. And yet how each gets there conceptually is quite different, and that difference matters a great deal from the standpoint of theory. As Turvey (2019) puts it:

Does perception create or constitute its own objects, so that the environment perceived by an organism depends on the organism’s act of perceiving? Or is the function of perceiving to acquaint an organism with the objects composing its environment as they exist independently of perceiving? (p. 29).

Occlusion transpiring at an edge serves as information that specifies an object going out of sight rather than going out of existence. Disocclusion at an edge, where an object gradually comes into sight, specifies an object previously hidden. Occlusion has a prospective quality; disocclusion a retrospective quality. This account is consistent with the claim that perception is direct; its qualities are specified by information that is available to be perceived, and one need not appeal to any mediating processes (e.g., a concept of object permanence that supplements perceiving).

An enaction explanation wouldn’t appeal to mediating processes either, and yet without grounding the phenomenon in specifiable information – information that can, in principle, be described with precision – the ‘enactment’ of currently hidden surfaces (i.e., sense making) seems nearly magical. At best, they are describing the phenomenology of the event, but because they begin with sensations, they miss the information that specifies it. After all, in the ecological account, occlusion at an edge transpires in the dynamic *relationship* between environment and perceiver, and what is perceived can be anchored in specifiable change in the ambient light, i.e., information-as-specification. Even if we take ‘enactment’ or sense-making as indicating an emergent event, *where* does it event occur? It would seem to be realized within the sensorimotor loop which is solely within the boundaries of each *individual* system. This difference illustrates one way that an ecological approach would embrace a direct realist stance and enaction theory would not. We will return to this implication in the final section of this paper where we discuss the matter of the organism’s boundaries.

### Conceptual Limitations of Ecological Optics From the Standpoint of Enaction Theory

Enactivists identify what they see as two related short-comings of the ecological approach to perceiving. Their first objection is that ecological psychology appears to take the perceived character of the world, or at least the information that specifies it, as “pre-given” in the environment prior to the presence of a perceiver. Presumably, at least part of the concern here is the apparent absence of an account why the environment appears as it does in the absence of the organism’s role in the act of perceiving. The second objection is that little is offered in the way of explaining what the individual contributes to perception – that merely stating that information is available to be detected or picked up by a perceiver is insufficient. Taken in combination, the claim is that without indicating how the individual contributes to perceiving, the available information would seem to be simply “pre-given,” and as a result perceptual experience would not have the transactional character – rather than a linear functional approach – that a dynamic approach to psychological functioning that *both* adopt would suppose. These criticisms initially offered by Varela et al. (1991) have continued to be repeated by advocates of enaction theory. They strike me as reflecting an inadequate understanding, first, of the relational character of ecological psychology; and, second and most critically, of the manner in which the environment is conceptualized from an ecological perspective (also, see Fultot et al., 2016).

### Is Information ‘Pre-given’?

Is the environment “pre-given” prior to the presence of a perceiver? If so, that would undermine the relational commitment of ecological psychology. In certain respects, this is a rather straight-forward question that can be answered in the negative. The very definition of an environment implies a possible animal. That definition is foundational to the ecological sciences and its notion of a habitat, and it is foundational to ecological psychology as well. Gibson begins his 1979 book by taking up “the environment to be perceived” (p. 5), and he defines the environment in the first sentence of Chapter 1 as “the surroundings of those organisms that perceive and behave, that is to say, animals” (p. 7). He draws the distinction between the “animal environment” and the physical world, with the latter referring to a domain taken independent of any animal, whereas “the words animal and environment make an inseparable pair” (p. 8). What *can be* an environment – rather than the physical world considered apart from animals – by definition implies a possible animal. The surface of the sun, for example, is part of the physical universe, but it is not an environment. Logically, an environment cannot pre-exist an animal because *what can be an environment* is defined in relation to an animal. Likewise, the information available to be perceived from the standpoint of ecological optics is to be taken relative to a possible perceiver.

In this same vein, the identification of a habitat implies a particular animal group or species. By definition, habitats are not empty slots to be filled by organisms, but they reflect the reciprocity of environment and a way of living. The environment does not pre-exist an animal when it is defined in relation to animal, as it is in the ecological sciences.

To indicate, however, that animal and environment ‘make an inseparable pair’ does not mean that in the case of perception – that is, when we are operating in the psychological domain – that the features of the environment cannot ‘exist’ independently of an animal in other respects. There is a particular sense in which an environment is ‘pre-given.’ These issues have long been points of confusion (see, Heft, 2001, pp. 132–135). How can features of the environment be independent of the individual at some times and also not independent given the mutuality of animal and environment? Consider the case of the chair in the next room that affords the possibility of sitting-on for me if I were to move from my desk chair where I am presently to that room. It is independent of me in the respect that it is in the next room; nothing that I do from here will affect it. But it only exists as an affordance possibility relative to me (or some other person). Contra Berkeley, ‘to be’ is not to be perceived; but rather ‘to be’ is to offer the possibility of being perceived by some individual for whom the environment is taken in relation. An affordance, such as the chair in the example, is defined relative to a prospective individual, but it is not necessarily perceived by that individual at all times. There are always places and features in the environment that are not necessarily in view at a given time. (See, for example, the discussion of sensation-less perceiving above.) The environment to be perceived is *an environment of possibilities* considered relative to a perceiver.

How are we to understand such cases? How does this state of affairs come about? Briefly, the environment, or better the habitat, exists separately from an animal’s actions and experience because the *histories* of each are different. This way of formulating the nature of perceptual experience can be found in William James’ philosophy of radical empiricism (James, 1912; also see Heft, 2001). Immediate experience stems from the intersection of processes in the environment and processes of the perceiver. Referring to the *immediate experience* of a room in which his reader might be located in, William James writes: “the experience is a member of diverse processes that can be followed away from it along entirely different lines. One of them is the reader’s personal biography, the other is the history of the house of which the room is a part. [That latter history includes] a lot of previous physical operations, carpentering, papering, furnishing, warming, etc” (pp. 173–174). Structure ‘on the environment side’ of relational, immediate experience (i.e., the perceiver-environment relation) is ‘already there’ available to be perceived when taken at the level of analysis of and in relation to a perceiver. Note that the environment, in this passage from James, is identified by following a set of relations away from immediate experience; that is, it is taken relative to the perceiver.

A particular place that an individual enters has already had a prior history that accounts for *why it is as it is* at the moment the individual encounters it (Heft, 2018). Its character is ‘already there’ for an individual who might encounter it; and yet only those features taken relative to a possible perceiver matter from a psychological standpoint. Let’s take the comparatively simple case of a building – and it is simple because we can have a complete understanding of its history. When an individual enters a building, its structure is indeed pre-given in the sense that it was already there upon entering. Why is that? Because designers, clients, contractors, among others, all had a hand in constructing it, and they did so with its intended purposes relative to possible users in mind. Needless to say, intentionally altering environments is an action that all organisms engage in for adaptive purposes (Odling-Smee et al., 2003). How the individual comes to detect the building’s structure, and in the end can find her way around in it as well as utilize its affordances, is a matter of exploration and discovery. Its potential structure taken relative to a perceiver is already available to be perceived. Environments like individuals have a history.

What is the case with a building, which is constructed by human efforts, is also the case with aspects of environments that bear less indication of human intervention – so-called ‘natural environments.’ Because our species lineage evolved in relation to particular features of the environment, such as ground surfaces and graspable objects, environments offer particular possibilities for action for *Homo sapiens*. In a sense these structures do pre-exist – they have a history – when considered relative to our species, but they don’t come into existence when a particular individual is present. They are ‘permanent possibilities’ for perceiving for an individual understood within the framework of ecological optics.

Gibson (1966) points out that the phrase ‘permanent possibilities’ for perceiving is a variation on John Stuart Mill’s hypothesis of the “permanent possibilities of sensation.” But the difference between these two phrases stems from Mill taking sensations as the starting point for an account of perceiving, as I suggest enaction theory does. As a result, “[h]e believed that their grouping constituted the basis for our *belief* in the external world, but this is far from asserting that the possibility of detecting stimulus invariance is the basis for *contact* with the external world’ (Gibson, 1966, p. 223). Does ‘belief’ in this context mirror enaction theory’s notion of sense-making?

### The Perceiver’s Contribution

Turning to the second objection cited above that ecological psychology gives short shrift to what the individual contributes to perception, Varela et al., 1991) argue that this approach “leads to a research strategy in which one attempts to build an ecological theory of perception entirely from the side of the environment” (p. 204). What is found to be missing in ecological psychology from the enaction perspective is a consideration of the organism’s role in perception. This is a common criticism of ecological psychology, although unlike most others, enaction theorists rule out that this role would involve providing non-perceptual mediating additions to the flow of sensory activity. Still, this criticism typically reflects a limited reading of Gibson’s writings, and in particular a cursory reading at best of Gibson (1966).

Most enactivists are familiar with ecological psychology through Gibson’s last book (1979) and later writings by other ecological psychologists; but the truly breakthrough work that launched ecological psychology is *The Senses Considered as Perceptual Systems* (Gibson, 1966). As its title indicates, that book reformulated perceiving conceptualized as a reception of stimulation and the imposition of sensations as instead a process whereby the individual engages an information-rich, structured environment through action. The visual perceptual system, for example, includes, in addition to the retinal receptors and the neural optic tract, the possibilities for action provided by the body (e.g., movements of the eyes, neck, and the entire body). Perceiving is an activity of the body, not merely the sensory tracts. Through perception-action, invariant and perspective structure is revealed that specifies environmental layout and self-movement. That is, *action generated by the individual* plays an essential role in revealing environmental structures, and in doing so supports exploration and discovery. That claim is central to the entire framework of ecological psychology. To assert that environmental psychology attempts to build an account of perceiving “entirely from the side of the environment” overlooks these essential facets of the framework invoked by the concept of a *perceptual system*.

Other and no less important ‘contributions’ on the perceiver side could be pointed to as well, such as changing attunement to available information through *perceptual learning and development*, and actions of directed attention on particular occasions. To take Gibson (1979) as reflecting ecological psychology in its entirety is to fail to take into account a great deal of other literature pertinent to ecological psychology not only by J. J. Gibson and those who followed him, but perhaps most especially, E. J. Gibson’s contributions. Her seminal book *Principles of Perceptual Learning and Development* (1969) – which in my view should be required reading for anyone interested in perception – as well as the later An *Ecological Approach to Learning and Development* (Gibson and Pick, 2001), are rarely mentioned by enaction theorists when criticizing ecological psychology in this vein. As Gibson (1966, p. 271) pointed out, the elaboration of their differentiation theory of perceptual learning (Gibson and Gibson, 1955) which is to be found in Gibson (1969) is an essential facet of the ecological approach. As he plainly indicated, that book “will take up the story where mine leaves off” (Gibson, 1966, p. viii).

Likewise, as Turvey (2019) recently stated about ecological optics, the hypothesis of information as specification “does not constitute a complete theory of perception. At this juncture, our concerns remain fairly modest and it suffices that we can identify a theory of direct perception as a theory whose distinguishing mark is the [information as specification] hypothesis” (pp. 30–31). What the availability of specifying information accomplishes, paraphrasing Turvey, is to negate the need for psychological factors that either transform or produce perceptual experience, *while still leaving ‘plenty of room’ for how psychological factors might affect how the organism ‘exploits’ available information*. Gibson consistently described the ecological approach as one whose development was on-going. This initial step of framing perceiving as the pickup of information takes us quite far epistemologically, because it would seem to undercut theories that assume perceptual experience to be mediated necessarily by non-perceptual processes, and hence necessarily indirect. But there remains much work to be done from a *foundation* of information-as-specification.

Recall that from the perspective of ecological psychology, perceiving supports adaptive functioning by making it possible for the organism to ‘stay in touch’ with the environment in the course of everyday actions. In the case of tactile perception or touching (as well as tasting), individuals can stay in touch with environmental structures proximally by means of literally feeling and manipulating surfaces. In order to stay in touch with the environment more distally, as is the case with seeing, hearing, and smelling, what is needed is a *medium* that carries structure specifying features at a distance from the perceiver. The essential place of the medium for perceiving from an ecological perspective has no counterpart in enaction theory. Presently, ecological optics is the most well-articulated framework to account for how it is possible that perceivers stay in touch with the environment through vision. Ecological optics and the concept of information-as-specification which requires a medium are critical to ecological psychology’s claim that perceiving even of features quite distant from the body surface is direct, that is, unmediated (For a discussion of the medium in ecological psychology and its antecedents, see Heft, 2001, pp. 225–232; also see, Nonaka, 2020).

In light of the enaction theory criticism of information in the ecological approach, we might ask inversely what makes a particular sensorimotor pattern informative from the enaction perspective? The results of sense-making must have some adaptive relation to the surround in which the system operates. That is, it must be meaningful in relation to the surround, or risk being disconnected from that surround and consequently have questionable adaptive value.

### What Makes Sensorimotor Activity Informative in the Enaction Approach?

Our previous discussion of enaction theory was mostly limited to a consideration of the ‘sensory’ facet of sensorimotor structures; and in doing so we omitted a central feature of the approach. To reiterate, proponents of enaction theory are clear that the sensory stream of activity alone is not the basis for perceiving the environment if for no other reason than actions of the individual cause changes in the sensory stream. Consider optic flow. The perceiver must have a means of distinguishing between changes in sensory activity due to occurrences in the environment, on the one hand, and sensory changes produced through self-action, on the other. Because there is sensory change in each case, i.e., change of retinal stimulation, what makes a particular instance of change *informative* as to its basis? Is it due to movement in the environment or movements of the eyes? From the enaction perspective, embedding sensations in a sensorimotor loop, with the latter considered in relation to a network of related sensorimotor linkages, results in one particular experience to be realized (enacted) for the individual rather than some other.

In order to understand what makes a particular sensorimotor loop *informative*, it must be considered relative to “the set of all possible sensory dependencies on motor states, for a particular type of agent and a particular environment. Whatever specific behavior the agent exhibits, its sensorimotor projections will always be found *within this set*” (DiPaolo et al., 2017, p. 53; emphases added). These authors call this set the ‘*sensorimotor environment*.’ However, we are cautioned that the sensorimotor environment, which would seem to be a state space within the system, “is not to be confused with the environment for the agent” – the latter presumably referring to the character of the surround beyond the boundaries of the system. (Because of this possibility for confusion, it might have been prudent to have utilized terms other than environment and, as we will see, habitat in these instances.)

They go on to distinguish between the ‘*sensorimotor environment’* and the “*sensorimotor habitat*.” Whereas the former refers to all possible trajectories in this state space, the latter refers to “the set of all sensorimotor trajectories (i.e., movements in a sensorimotor space) that can be generated” *by a particular age*nt (p. 54). To do this, one must take into account “internal dynamics’ of the perceiver, in addition to constraints from the sensorimotor environment. “In other words, although the SM environment limits the possible [SM] habitats, there are still an infinite number of ways in which an SM environment can be ‘inhabited”’ (DiPaolo et al., 2017, p. 54). Without going into further detail here, which would require a discussion of, e.g., “sensorimotor coordination” and “sensorimotor schemes” (see, pp. 55–58), it will be useful to examine these claims more closely in order to understand what makes any particular sensorimotor activity informative from this perspective. In other words, what makes sensorimotor activity A indicative of circumstance X (e.g., eye movement) or Y (e.g., movements in the environment) in that the visual ‘sensations’ are assumed *the same* in each case?

Any particular instance of a sensorimotor activity is informative relative to that set which could be generated by a particular agent. To return to the object property of sponginess, DiPaolo et al. (2017) ask us to imagine that one is “a worker whose daily task is to sort out 1000s of sponges into soft and hard” (p. 61). As I understand their argument, the present sponge can be identified as soft or hard because of the amount effort employed to depress it and reciprocally the force of return resistance one experiences through tactile sensations *in relation to a prior set of possible sensorimotor patterns*. The present sponge is experienced, for example, as soft but not hard; and it is so because it is informative as to “what it is not but might have been.” That latter phrase comes from Gibson (1966, p. 245) brief discussion of the concept of information as it is employed in communication theory developed by Shannon (1948). Gibson’s discussion here was an effort to clarify the difference between his concept of information (what we called above following Turvey ‘information-as-specification’) and information as developed by Shannon. Gibson regularly expressed frustration that some critics confused the two. Shannon’s concept of information was adopted and applied to the psychology of perception, most prominently by Attneave (1959) and Garner (1962).

I have not found any reference to Shannon’s ideas in my admittedly selective examination of the enaction literature; but the manner in which DiPaolo et al. (2017) treat the role of sensorimotor activity in sense-making – e.g., the realization of one sensorimotor pattern within a set of sensorimotor trajectories – appears to be similar to that line of thought. To explain, Shannon’s influential treatment of information was developed post WWII for the field of electronic communication The prototypical problem for communication theory is how a signal transmitted by a ‘sender’ that is informative of something, as opposed to, e.g., mere ‘noise,’ can be recognized by a ‘receiver.’ Imagine an individual (sender) speaking into the microphone of a telephone, and a listener (receiver) camped out at the other end of the line. Absent any pre-established background structure in the receiver relative to the signal being sent (e.g., the receiver doesn’t speak the same language as the sender), the signal would be uninformative. But with some pre-established background structure on the part of the receiver, a signal can be recognized as being informative at the receiver end. According to Shannon information theory, this is done not by registering the signal as such because, as we saw in the case of a language, some background is needed on the receiver side of things. Instead, the signal is rendered as being informative by ruling out what it isn’t with respect to some prior background (i.e., what is already known.) What makes a signal informative is that it rules out *prior* possibilities as to what it could be. In other words, and critically, that which is informative reduces uncertainty for an individual.

How are we to understand this difference in how ‘what is informative’ is treated in ecological psychology and enaction theory? We already tried to ground information qua ecological psychology in an analysis of structure through ecological optics taken in relation to a perceiver. What about the enaction approach? The latter like most 20^th^ century perception theories appears to assume that organisms operate in a psychological domain of possibilities. The sensorimotor environment is the set of “sensorimotor projections” within which a particular behavior exhibited by an agent on any particular occasion will be found. This set is narrowed further when the individual’s “internal dynamics” are added into the account, resulting in a sensorimotor habitat. Perceiving qua sense-making appears to be realized in relation to this background set of possibilities. What is perceived is realized among a set of other different possibilities within a particular domain.

(Although it is not to be minimized, we set aside here the important question of the origin of the background possibilities – the SM environment – against which a particular sensorimotor correspondence is assessed. If the informative character of a particular sensorimotor activity is established relative to a field of possibilities, how does that field of possibilities itself come about? One cannot merely assume that the necessary background for rendering an instance of sensorimotor activity is in place without explaining its origins. Articulating the requisite developmental history is needed. I cannot speak to how adequately enaction theory carries this out.)

In contrast, and as we saw above, from an ecological perspective, the perceiver engages a surround that, in principle, has infinite nested informational structure; and continuing exploration may reveal ever greater subtleties or higher-order patterns in any particular domain. On-going discovery of ever finer structures is, for example, the basis for connoisseurship in some area of activity. The training of a sommelier (a wine expert), for instance, consists of hours and hours of comparative wine tasting, in the course of which distinctive relational differences *in* the chemical make-up of the wine are revealed to the perceiver. To reiterate, the differences are to be found *in the wine*, and reciprocally, with continued exploration, the taster’s acuity is heightened. As Gibson (1969) proposed, what differentiates A from B is a relational difference, and that difference is discoverable through exploration of the array of available information.

For their part, enaction theorists criticize ecological psychology on the grounds that “*the origin* of the particular motor patterns that bring about the invariant revealing transformations is not always considered relevant; instead, what matters in many case is simply *the structure of movement-induced flows*” (DiPaolo et al., 2017, p. 74, emphasis added). But, to continue the example above, acts of discovery through wine-tasting do not involve merely imbibing and allowing the movement of liquid to flow across receptors surfaces. Swirling the wine in one’s mouth and other acts of exploration in order to reveal its informational structure are critical (Gibson, 1966, pp. 138–144). The actions are relevant for the purposes of perceptual differentiation and discovery, but “origins of the particular motor patterns” in this case would not seem to be.

What accounts for the route taken in enaction theory, or at least, by DiPaolo et al. (2017)? Why treat a given sensorimotor pattern as being informative relative to a set of possibilities? My supposition is that it is traceable to the concern raised in the first part of this paper – their reliance on sensations, even when embedded in sensorimotor activity, on which to develop an account of perception. Sensations tend to be equivocal in relation to their environmental sources. The environment, as a consequence, is in principle an unknowable Kantian “thing-in-itself.” But an unknowable world for Kant was world of apart from human experience, and a pre-Darwinian one at that – not a habitat from the point of view of the ecological sciences with which organisms need to ‘stay-in-touch.’ The best one can do from an enaction stance is to ‘enact’ a perceived world through sense-making, but that would seem to put the perceived world ‘within’ the organismic system.

### System Boundaries and the Region of Exchange

One of the requirements that marks agency in a biological structure, according to DiPaolo et al. (2017), is “system individuation.”

“The enactive approach suggests that agents are systems that *actively define themselves as individuals* and may be identified as such without arbitrariness. Only systems that manage to sustain themselves and distinguish themselves from their surrounding, and in so doing define an environment in which their activity is carried out, are considered as candidate agents in this approach” (p. 112).

This point of view is certainly one that would be congenial to the ecological psychology perspective, although the way it is further developed within each approach adds to a divide between them.

To account for system individuation, DiPaolo et al. (2017) invoke the notion of *operational closure*, which refers “to a network of processes whose activity produces and sustains the very elements that constitute the network” (p. 112). The interdependencies among the system’s constituents give rise, as a matter of course, to a boundary that distinguishes this network of interrelations from those things that lie outside of it. “[T]he boundaries between an autonomous system and its environment emerge as a result of how an autonomous system is organized” (DiPaolo et al., 2018, p. 27).

That said, it is obvious to enaction theorists that the system cannot be wholly independent of the surrounding environment. Because it is a far-from-equilibrium system, it must remain sufficiently open to the surround to allow needed resources beyond its boundaries to participate in the necessary workings of the system. At the level of cells, we find semi-permeable membranes that allow, e.g., nutrients to enter into the system to participate in and maintain its functions. Critically for our purposes here, there is *region of exchange* between the environment and the system; and that region is where the organism ‘ends’ and the environment ‘begins.’ In this biological case of the cell membrane, that region of exchange is *proximal* – at the physical body boundary.

When we shift our level of analysis from the cell to the organism as a whole, and adopt a higher-order *psychological* focus, we also find some instances of a proximal region of exchange between the organism and the environment, as in the case of tactile perception (see above). But also, and particularly striking, are those commonplace experiences when *the region of exchange* between the organism and the surround is experienced as being located at places distant from the physical body boundary. In those cases, the body is experienced as being extended *distally* into the environment.

To offer two obvious examples: when individuals use a tool, such as a stick to probe a surface or a screwdriver to tighten a screw, they invariably report that the environment is experienced as beginning at the end of the tool – at the surface and at the screw notch, respectively – and that *the body is experienced as if it extends to that point.* Exchanges with the environment as mediated by tools are typically reported as being felt at some distance from the biological boundary of the body.

With their roots partially in phenomenological writings, both ecological psychology and enaction theory recognize this phenomenon. But how readily can each approach begin to account for it? DiPaolo et al. (2017) differentiate in an *ad hoc* manner among different levels of complexity of sensorimotor agency, from those of “minimal sensorimotor engagement,” such as habits, to “open sensorimotor agency” whereby “sensorimotor agents have the adaptive capacity to learn new sensorimotor schemes and integrate them in the overall network” (p. 170). This latter integration would seem possible in their account because the system is open to temporary ‘enabling relations’ with factors outside of it. Notably, however, *these factors do not enter into the system’s interdependencies*. Although they “do not belong to this network [they] enable processes within the network; but they remain external because they themselves do not depend for their operation on processes in the system” (DiPaolo et al., 2018, p. 25).

However, if these temporary enabling relations do not belong to the network of system interdependencies, how can an enaction account of perception go beyond the boundaries of the system in the ways that *the experience of tool use* suggests? As we just saw, enabling relations only serve “to enable processes within the network.” Can these enabling relations function to extend the network experientially? Presumably, it is assumed that they can, but how they might do so is not explained. Most important for our discussion, by setting ‘enabling relations’ outside of the network system, the primary unit of analysis that is in evidence here is the individual system rather than an organism-environment relation.

An alternative way of conceptualizing the experience of the extended body that accompanies tool use is to adopt a relational view with the organism-environment as our unit of analysis. The network of interdependencies at work would encompass the organism and the environment as coupled features on any particular occasion. The animal-environment relation is the unit of analysis for ecological psychology; and it is characteristic of the focus inherent in the ecological sciences rather than biology as such.

The study of ecology takes the organism in relation to a system of environmental interdependencies. Enaction theory while recognizing the tight interdependencies within the organismic system seems to underplay the interdependencies of an organism-environment system. Conceptually in the ecological sciences, what is needed from the standpoint of an organism’s functioning is a means of ‘staying in touch’ with circumstance in its habitat. Over the course of species’ evolution, perceptual systems were selected for in order to make this possible. These perceptual systems offered a selective advantage for organisms because by means of them, they could exploit the information that was available in the surround in order to maintain the necessary *coupling* between on-going environmental change and dynamic organismic processes.

Turvey (2019) points out: “what makes a theory of perception a theory of direct perception at its most fundamental level is an enriched entailment structure” (p. 40). By the latter he means an account of the information available to be detected that specifies the environment. In spite of enaction theory allowing for so-called ‘enabling relations’ between the system and factors external to it, in the absence of an account of the environment from an ecological perspective, and available information to specify the environment, is hard to imagine how the enaction approach can account for the experience of the extended body other than merely stating that it is enacted, and leaving the matter at that. A *coupling* between on-going environmental change and dynamic organismic processes requires an entailment structure on the side of the environment, as just noted.

Holt (1931), a student of William James and one of Gibson’s graduate school mentors (Heft, 2001) proposed that every action of an organism has a quality of ‘adience’ by which he meant the quality of ‘reaching toward’ a source of stimulation – that is, it has a quality of external reference. That sort of intentional stance is shared by both enaction theory and ecological psychology. But Holt goes a step farther and helps set the stage for the relational stance of ecological psychology by arguing that these actions can only be adequately described with reference to factors outside of the organism beyond the body boundaries (Holt, 1915). In this way distant features function as *constituents* of action. They are enfolded within the action itself. According to Holt, it is only when we recognize the adient quality of action that we are led *from the study of biological processes to the study of psychological functions* (Heft, 2014).

Arguably, however, sensory activity (in sensorimotor activity) cannot conceptually ‘carry’ the properties of ‘distant objects’ (see above), and for this reason, those ‘distant objects’ cannot function as ‘constituents of the behavior process’ from an enaction perspective. Organismic processes accordingly seem to remain encapsulated within system boundaries. In contrast, Holt offers a relational perspective: “The knower is a concrete material body in a concrete material environment and the cognitive relation exists between the two” (Holt, 1931, p. 51).

A relational view requires an account of ‘both sides’ of the environment-person duality. In Gibson’s ecological approach, his formulations of ecological optics, on the one hand, and perceptual systems, on the other – both of which are introduced in Gibson (1966) – make a relational perspective for psychology feasible. An account of the potential ‘environment’ side of the relation allows us to take our *psychological* analysis beyond the organism considered solely with respect to its *biological* boundaries.

## Concluding Comments

It has been argued that ecological psychology and enaction theory, in spite of their considerable commonalities, remain at variance because of differences over several basic issues: first, the role that sensations play within each approach differs, with enaction theory taking these products of receptor activity to be the initial basis for ‘contact’ with the surround, while ecological psychology considers sensations as being incidental at best for perceiving.

Second, they differ in the manner in which each treats the concept of information, with enaction theory seemingly conceptualizing information in a manner similar to that employed in Shannon’s account of communication, with its model being a sender and a receiver in possession of possible states of realization; while information within ecological psychology refers those structures in the medium for perceiving that specify properties and features of the environment (i.e., information-as-specification). The latter meaning of information stems from Gibson’s development of ecological optics. Criticisms of ecological psychology from the point of enaction theory often reflect a failure to recognize the place of ecological optics in this approach.

Third, the way in which each approach conceptualizes the organism’s boundaries differ. Enaction theory’s focus on ‘system individuation’ gives rise to an emphasis on the organism’s boundary that distinguishes its network of interrelations from those things that lie outside of it. This image stems from considerations at a biological level of analysis such that individual systems, such as a cell, while dependent on exchanges with the surround, are considered mostly apart from it. Ecological psychology adopts as its unit of analysis the organism-environment relation in keeping with the orientation of the ecological sciences. From that perspective, the boundary or region of exchange between the organism and the environment – that is, where the organism ‘ends’ and the environment ‘begins’ – is fluid on functional and psychological grounds.

The common thread running through all of these differences is, in fact, the first one – the place of ‘sensations’ in ecological psychology and enaction theory. Recall that it was the conceptual inadequacies of sensations for developing an account of perception that eventually led Gibson to reject sensation-based approaches to perception and in their stead to offer an information-based approach. From this historical vantage point, enaction theory in spite of its valuable incorporation of new ideas from dynamical systems thinking for perceptual theory, brings along with it old ideas that have not served perceptual theory particularly well in the past.

## Author Contributions

The author confirms being the sole contributor of this work and has approved it for publication.

## Conflict of Interest

The author declares that the research was conducted in the absence of any commercial or financial relationships that could be construed as a potential conflict of interest.

## Footnotes

1 https://www.youtube.com/watch?v=6QXqz_UJPWM
